# Indocyanine green fluorescence in parathyroidectomy: enhancing efficiency through real-time adenoma identification

**DOI:** 10.1007/s00405-025-09679-0

**Published:** 2025-11-01

**Authors:** Yuval Mizrakli, Zeev Hirschfeld, Jacob Pitaro, Haim Gavriel, Limor Muallem‑Kalmovich

**Affiliations:** 1 Department of Otolaryngology-Head and Neck Surgery, Shamir Medical Center (formerly Assaf Harofeh Medical Center), Zerifin, Israel; 2 Gray Faculty of Medical and Health Sciences, Tel Aviv University, Tel Aviv, Israel

**Keywords:** Hyperparathyroidism, Parathyroidectomy, Parathyroid adenoma, Indocyanine green (ICG),, Near-infrared (NIR) imaging, Intraoperative imaging

## Abstract

**Purpose:**

Intraoperative identification of parathyroid adenomas can be challenging due to their anatomical variability and the limitations of preoperative imaging. This study aimed to evaluate the impact of indocyanine green (ICG) fluorescence imaging on operative efficiency and surgical outcomes in parathyroidectomy, employing a standard near-infrared (NIR) endoscopic system.

**Methods:**

We conducted a prospective interventional study with retrospective controls. Patients undergoing parathyroidectomy for primary hyperparathyroidism were included. The study group received intravenous ICG for intraoperative fluorescence imaging to aid parathyroid gland identification. Standard protocols, including preoperative imaging, intraoperative quick parathyroid hormone (qPTH) measurements, and frozen section analysis, were followed in both groups. Operative times and clinical outcomes were compared between ICG-assisted and standard procedures.

**Results:**

Seventy-six patients were included: 19 in the ICG group and 57 in the control group. The median net operative time (cutting-to-end) was significantly shorter in the ICG group (59 vs. 79 min; *p* = 0.002), while entry-to-cutting time was slightly longer (44 vs. 35 min; *p* = 0.014). Although the total operative time was shorter in the ICG group (109 vs. 123 min), this difference was not statistically significant (*p* = 0.060). A ≥ 50% reduction in qPTH was achieved in 94.7% vs. 89.5% (*p* = 0.672), and adenoma confirmation was 100% vs. 96.5% (*p* = 1.0), respectively.

**Conclusion:**

ICG fluorescence is a cost-effective adjunct to standard parathyroidectomy, offering real-time gland visualization and potentially reducing operative times. Its integration into the routine surgical workflow may enhance intraoperative efficiency and outcomes.

## Introduction

While parathyroidectomy is often straightforward when adenomas are preoperatively localized, intraoperative identification may still pose significant challenges—even for experienced surgeons [1]. The parathyroid glands can exhibit highly variable anatomical locations, especially in cases of ectopic, intrathyroidal, or multiglandular disease [2, 3]. Preoperative imaging modalities such as technetium-99 m sestamibi (MIBI) scintigraphy, ultrasound, and 4D computed tomography can assist in localization, but their sensitivity and specificity are variable [4–8]. Even when they are concordant, these imaging results may not reliably correspond to intraoperative findings, posing challenges for surgical planning and decision-making [9–12].

While effective, current intraoperative methods for confirming parathyroid tissue—such as frozen section analysis and quick parathyroid hormone (qPTH) measurements—are time-consuming and not universally available, potentially prolonging surgery and limiting access to optimal care [13–16]. The need for real-time, efficient intraoperative tools remains unmet.

Indocyanine green (ICG) fluorescence has emerged as a promising adjunct in endocrine and neck surgeries [17]. ICG is an FDA-approved, water-soluble dye with a favorable safety profile, which emits fluorescence under near-infrared (NIR) light [18, 19]. When administered intravenously, it binds to plasma proteins and remains within the vascular system, producing fluorescence when excited by NIR light [20]. This signal is detected by specialized imaging systems equipped with NIR cameras. The vascularity of parathyroid glands allows for strong fluorescence signals, enabling surgeons to visualize and distinguish them from adjacent tissues [21]. The technique has been increasingly adopted in endocrine and neck surgeries for identifying parathyroid glands and assessing their perfusion, with additional applications in cardiovascular, hepatobiliary, and reconstructive procedures [22, 24, 25].

In addition to ICG-based methods, parathyroid glands exhibit intrinsic NIR autofluorescence, which enables real-time detection without exogenous dyes. This property arises from naturally occurring fluorophores within the gland, enabling distinction from surrounding tissues. However, autofluorescence intensity may vary between patients—and even between individual glands in the same patient. Additionally, specialized NIR imaging devices are often costly, and signal quality may be affected by ambient lighting or reflective instruments [26].

Despite its growing use, there remains limited evidence on the impact of ICG fluorescence on operative times and clinical outcomes during parathyroidectomy. Our primary objective was to evaluate the utility of ICG fluorescence for identifying parathyroid adenomas and to compare operative times and clinical outcomes between ICG-assisted and standard parathyroidectomy.

Furthermore, various optical devices are used to evaluate fluorescence intensity in the target tissue and are categorized as either image-based or probe-based. Devices such as the PTeye™ (Medtronic^®^) and FLUOBEAM LX (Ionmed^®^) are available but have not been widely adopted due to cost considerations. In contrast, the Olympus VISERA ELITE II Infrared Imaging System, when paired with a standard 0-degree endoscope, is widely accessible, as it is routinely used in general, gynecologic, and pediatric surgeries. Our secondary objective was to assess the feasibility and potential added value of using this widely available endoscopic fluorescence system during parathyroidectomy.

## Methods

This study was a prospective interventional study with retrospective controls, designed to evaluate the impact of intraoperative ICG fluorescence on parathyroidectomy outcomes. The study was approved by the Institutional Review Board (IRB), and informed consent was obtained from all participants in the intervention group.

Inclusion criteria consisted of individuals undergoing surgery for primary hyperparathyroidism, with preoperative imaging using MIBI scintigraphy and ultrasound. Exclusion criteria included previous neck surgeries, as well as conditions contraindicating ICG use, such as allergy to iodine or iodinated contrast agents, severe liver dysfunction given the hepatic metabolism of ICG, and a history of hypersensitivity to ICG or its excipients. The ratio of controls to ICG-assisted cases was 3:1.

ICG and endoscopy were not used as primary localization tools; but rather intraoperative confirmation following careful dissection and following visual and anatomic identification of the adenoma. All ICG-assisted surgeries were performed by a single experienced head and neck surgeon, following standard parathyroidectomy protocols. Intraoperative peripheral qPTH levels were obtained at baseline and after post-excision to confirm biochemical success. Frozen section was used for intraoperative confirmation when indicated. Control group procedures were conducted by the same surgeon or two additional senior head and neck surgeons under similar conditions, but without ICG utilization. The two groups were defined chronologically, before and after ICG implementation. A sensitivity analysis demonstrated no significant differences in baseline characteristics or outcomes between the senior surgeon of the study group and the other surgeons among the control group.

**Fig. 1:**
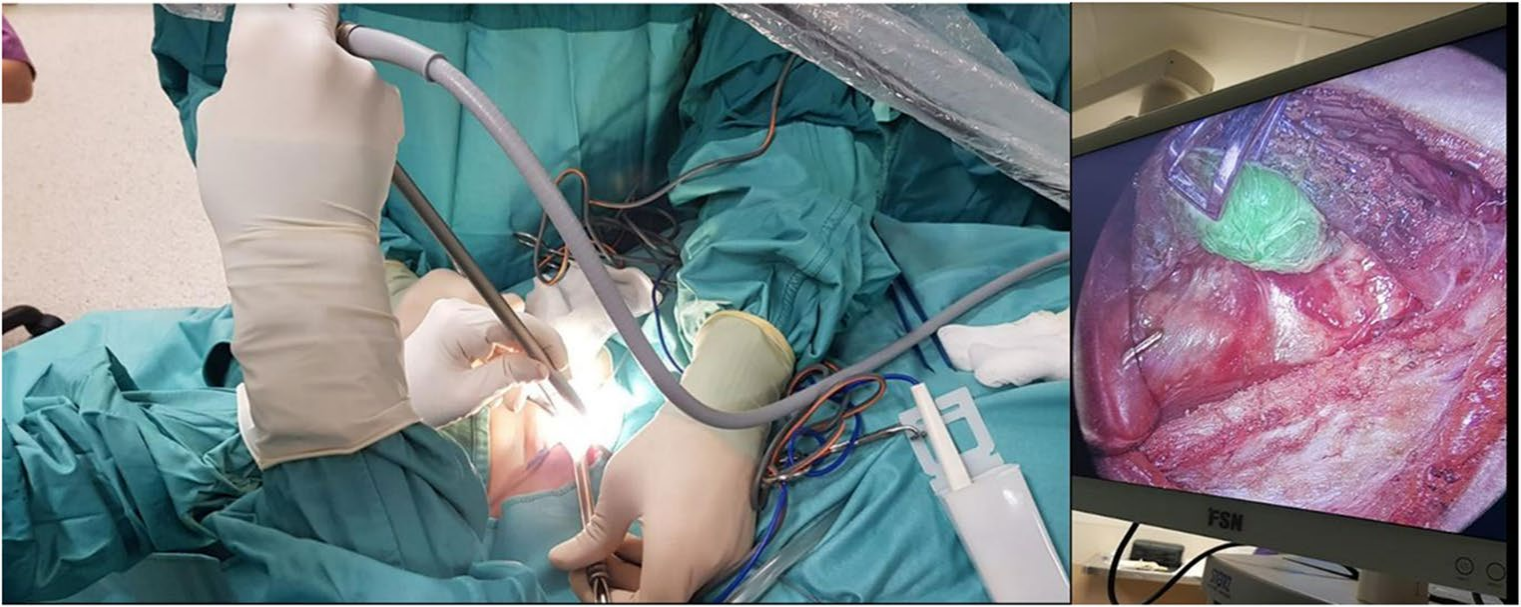
Intraoperative setup for the indocyanine green (ICG) near-infrared (NIR) fluorescence imaging system

After exposure of the thyroid and parathyroid regions, identification of parathyroid tissue in the ICG group was performed visually and with the aid of intraoperative adjuncts. ICG (fluorophore ICG-Pulsion^®^, Diagnostic Green GmbH, Aschheim-Dornach, Germany 25 mg) was prepared as a 7.5 mg dose (3 ml after dilution in 10 ml of sterile water) and administered intravenously five minutes prior to gland dissection. Fluorescence imaging was performed using the Olympus VISERA ELITE II Infrared Imaging System with a standard 0-degree endoscope (Fig. 1), allowing real-time visualization of parathyroid adenomas. Fluorescence signals were recorded to distinguish parathyroid adenomas from adjacent tissues.

**Fig. 2:**
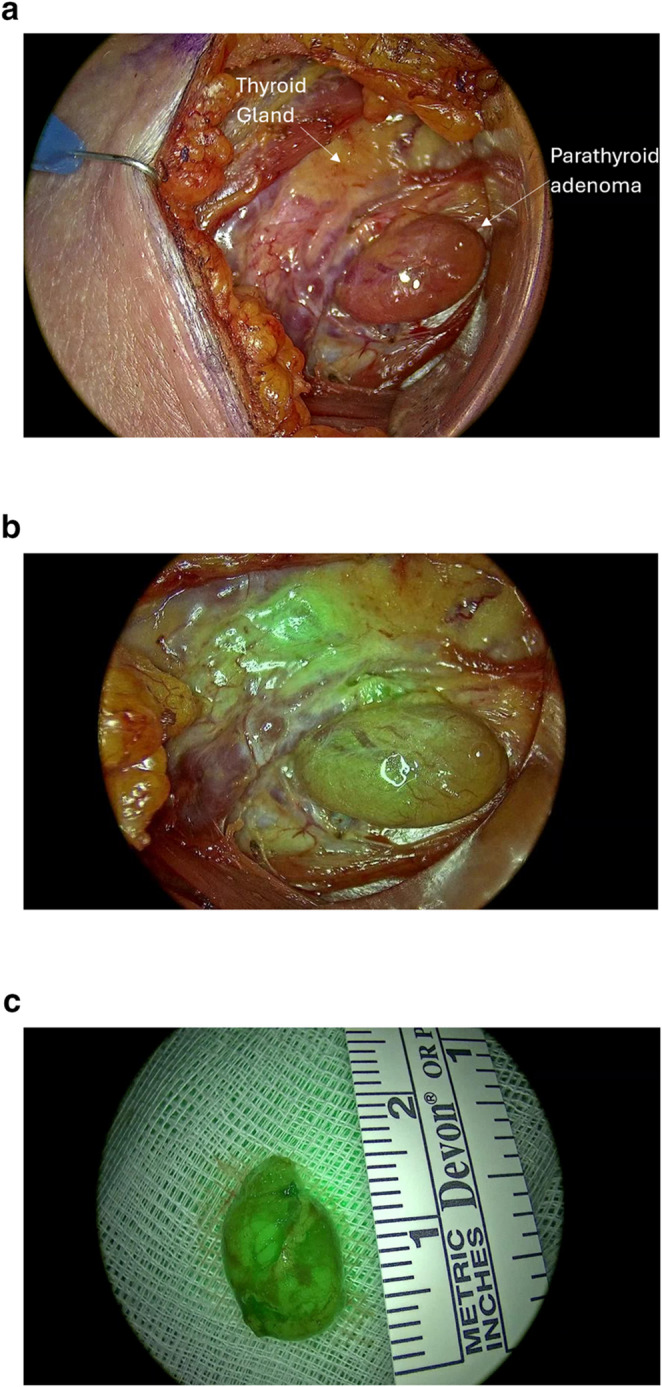
(**a**) Standard endoscopic view of a parathyroid adenoma in a second patient prior to ICG injection (**b**) In-vivo near-infrared ICG fluorescence imaging in the same patient, demonstrating enhancement of the parathyroid adenoma relative to adjacent structures (**c**) Ex-vivo ICG fluorescence of the excised parathyroid adenoma, confirming retained signal intensity and successful tissue identification

**Fig. 3:**
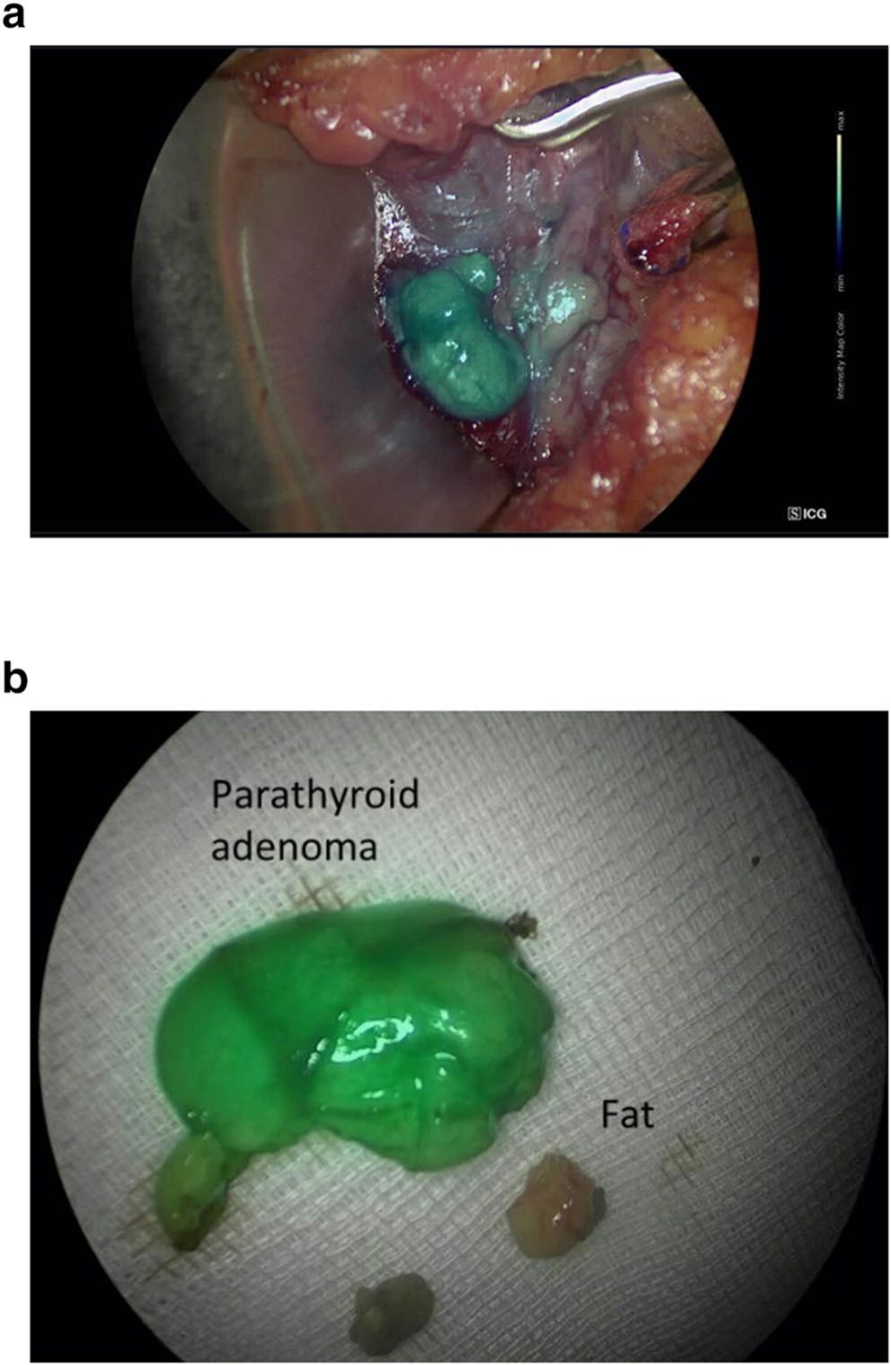
(**a**) In-vivo ICG fluorescence imaging showing enhancement of a parathyroid adenoma located on the carotid artery, with the thyroid gland retracted. (**b**) Ex-vivo ICG fluorescence imaging of the same patient’s resected parathyroid adenoma compared with excised fat

Intraoperative imaging included both in-vivo and ex-vivo assessments. Initial identification of parathyroid adenomas was based on visual and anatomical landmarks using standard endoscopy (Fig. 2a). Following intravenous ICG administration, in-vivo fluorescence imaging demonstrated clear enhancement of the target glands, aiding in differentiation from surrounding structures such as thyroid tissue and fat (Figs. 2b and 3a). Ex-vivo fluorescence of the resected specimens confirmed signal retention consistent with parathyroid tissue (Figs. 2c and 3b), supporting intraoperative findings.

Outcome measures included the accuracy of ICG fluorescence in identifying parathyroid adenomas, confirmed histologically, and operative times (entry-to-cutting, cutting-to-end, and overall). Secondary outcomes included the proportion of patients achieving a ≥ 50% intraoperative qPTH reduction.

### Statistical analysis

Continuous variables, such as operative times, were compared using the Mann-Whitney U test and are presented as medians with interquartile ranges (IQRs). Categorical variables were analyzed using chi-square or Fisher’s exact test where appropriate and are reported as absolute numbers and percentages (n, %). A *p-value* of < 0.05 was considered statistically significant. Statistical analysis was performed using SPSS version 25 (IBM Corp., Armonk, NY, USA). Graph was created using GraphPad Prism version 9 (GraphPad Software Inc., San Diego, CA, USA).

## Results

Seventy-six patients were included: 19 underwent surgery with intraoperative ICG fluorescence, and 57 underwent standard parathyroidectomy without ICG. All patients were followed postoperatively for a minimum of six months. Median age was 62.0 years (IQR: 54.3–72.8) in the ICG group and 63.6 years (IQR: 47.0–73.0) in the control group (*p* = 0.986). Female predominance was observed in the ICG group (16 patients [84.2%]) compared to the control group (35 patients [61.4%]), although this did not reach statistical significance (*p* = 0.067). Preoperative imaging demonstrated high MIBI–ultrasound concordance in both groups: 18 patients (94.7%) in the ICG group and 52 patients (91.2%) in the control group (*p* = 1.0). Other characteristics were similar between groups, including side of surgery (right side: 47.4% vs. 45.6%; *p* = 0.894) and gland diameter on imaging (12 mm [IQR: 9–18] vs. 14 mm [IQR: 10–17.5]; *p* = 0.672) (Table 1).

**Table 1:** Patient and preoperative characteristics

	ICG *n* = 19	Controls *n* = 57	*P* value
Age (years) *median (IQR)*	62.0 (54.3–72.8)	63.6 (47.0–73.0)	0.986
Female sex *n (%)*	16 (84.2%)	35 (61.4%)	0.067
Right side *n (%)*	9 (47.4%)	26 (45.6%)	0.894
Diameter per imaging in mm *median (IQR)*	12 (9–18)	14 (10-17.5)	0.672
MIBI-US agreement *n (%)*	18 (94.7%)	52 (91.2%)	1.0

Abbreviations: *ICG* indocyanine green, *IQR* interquartile range, *MIBI* technetium-99 m sestamibi, *US* ultrasound

**Fig. 4:**
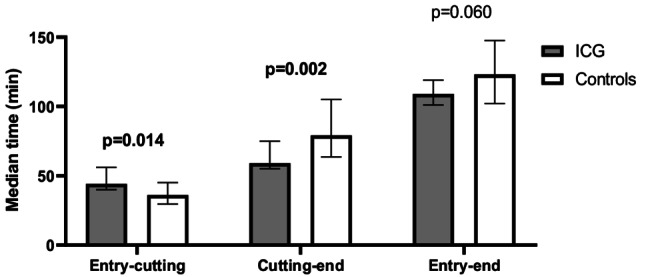
Operative time distribution in ICG and Control Groups. Median operative times (minutes) are shown for each surgical phase. Error bars indicate interquartile ranges

Use of ICG was associated with differences in operative times. The entry-to-cutting time was significantly longer in the ICG group, with a median of 44 min (IQR: 40–53) compared to 35 min (IQR: 28–43) in the control group (*p* = 0.014). The net operative time (cutting-to-end) was significantly shorter in the ICG group at 59 min (IQR: 55–75) compared to 79 min (IQR: 64–104) in the control group (*p* = 0.002). Although the total entry-to-end time was lower in the ICG group (109 min [IQR: 101–119]) compared to the control group (123 min [IQR: 102–147]), this difference was not statistically significant (*p* = 0.060) (Fig. 4).

In terms of surgical outcomes, a ≥ 50% reduction in intraoperative parathyroid hormone (qPTH) levels was achieved in 18 patients (94.7%), compared to 51 patients (89.5%) in the control group (p= 0.672). Pathological confirmation of adenoma was obtained in 19 patients (100%) in the ICG group and 55 patients (96.5%) in the control group (p= 1.0). The median weight of resected specimens was 863 mg (IQR: 300–1900) in the ICG group and 561.5 mg (IQR: 410–880) in the control group, with no statistically significant difference (p = 0.363) (Table 2).

**Table 2:** Operative and clinical outcomes

	ICG *n* = 19	Controls *n* = 57	*P* value
QPTH decreased by ≥ 50% *n (%)*	18 (94.7%)	51 (89.5%)	0.672
Pathology specimen weight (mg) *median (IQR)*	863 (300,1900)	561.5 (410,880)	0.363
Adenoma per histology *n (%)*	19 (100%)	55 (96.5%)	1.0

Abbreviations: *ICG* indocyanine green, *NIR* near-infrared, *IQR* interquartile range

## Discussion

This study demonstrates the utility of ICG fluorescence, combined with a standard endoscope, as an effective adjunct for intraoperative identification of parathyroid adenomas— leading to enhanced visualization and reduced operative duration. Additionally, the study showed high rates of qPTH reduction and adenoma confirmation in the ICG group, supporting the clinical utility of this technique. Unlike specialized autofluorescence cameras or probe-based devices, which are costly and dedicated solely to parathyroid identification, the endoscopic fluorescence system used in this study is widely available in surgical settings. When combined with inexpensive ICG ampoules, this approach may offer a practical and affordable solution.

Our findings align with previous studies that highlight the advantages of ICG fluorescence imaging in endocrine and neck surgeries [27–30]. Prior reports have demonstrated its utility the identification and preservation of parathyroid glands, particularly in challenging cases of ectopic or intrathyroidal glands [31, 32]. These results support the growing evidence of ICG as a reliable and practical tool for enhancing parathyroid identification and optimizing surgical outcomes. Parathyroid autofluorescence has also gained attention as an alternative to ICG-based fluorescence imaging. However, parathyroid adenomas often exhibit variable autofluorescence patterns, with adenomatous tissue demonstrating weaker fluorescence than surrounding normal parathyroid tissue [33, 34]. Such variability complicates intraoperative identification and may limit the utility of autofluorescence in real-time surgical settings. By contrast, the more consistent and robust signal provided by ICG underscores its advantage over intrinsic autofluorescence, particularly when autofluorescence intensity is unreliable.

In addition to its diagnostic value, ICG fluorescence proved intuitive and easily integrated into the surgical workflow, with minimal disruption. Although the learning curve is relatively short, outcomes may still be influenced by surgeon experience and familiarity with the imaging system. The results of this study underscore the potential of ICG fluorescence imaging to improve intraoperative decision-making and enhance surgical efficiency. By offering real-time visualization of vascular-rich parathyroid glands, ICG reduces the need for extensive dissection and tissue differentiation efforts. This is particularly advantageous in complex cases, such as ectopic gland locations. However, ICG is most effective in visualizing single, well-perfused adenomas and may not consistently identify multiglandular disease.

Postoperative calcium homeostasis is another important consideration following parathyroid surgery. While our study did not directly aim to evaluate calcium trends, serum calcium levels were routinely monitored during hospitalization. None of the patients experienced clinical hypocalcemia or required intravenous calcium supplementation. Moreover, long-term follow-up did not reveal cases of recurrent hypercalcemia, suggesting durable biochemical control.

Additionally, although our cohort focused on primary hyperparathyroidism, the use of ICG fluorescence may be considered for future applications such as identifying parathyroid glands post autotransplantation. However, this concept remains exploratory and warrants further study.

Furthermore, ICG fluorescence may reduce reliance on intraoperative qPTH measurements, which are both costly and time-consuming [35–37]. The ability to obtain real-time visual feedback during surgery helps streamline workflow, resolve intraoperative uncertainties, and shorten operative duration—all contributing to improved cost-effectiveness.

This study has several limitations. The relatively small sample size and single-center design may limit the generalizability of the findings. Moreover, although a sensitivity analysis revealed no significant differences between surgeons, the fact that all ICG cases were performed by a single surgeon may limit extrapolation to broader surgical settings. Future studies should include larger, multicenter cohorts and randomized designs to better establish the efficacy and generalizability of ICG fluorescence.

Further research is warranted to explore the application of ICG fluorescence in more complex surgical scenarios, including re-operative parathyroidectomies or cases involving multiglandular disease. Studies evaluating the long-term cost-effectiveness of ICG, particularly in reducing the reliance on intraoperative qPTH measurements and reducing hospital stay, would also be valuable.

## Conclusion

This study highlights the potential benefits of ICG NIR fluorescence endoscopy in improving the efficiency and precision of parathyroidectomy. ICG imaging provides real-time, reliable, and cost-effective visualization of parathyroid glands, helping to streamline surgical workflow and reduce operative times. While not intended as a stand-alone diagnostic tool, ICG serves as a valuable adjunct to traditional surgical identification methods and may reduce reliance on intraoperative techniques such as qPTH measurements. Its ease of integration with existing endoscopic systems further supports its utility as a practical tool in routine parathyroid surgery.

## Data Availability

The datasets generated and analyzed during the current study are available from the corresponding author on reasonable request.

## References

[1] Noltes ME, Cottrell J, Madani A, Rotstein L, Gomez-Hernandez K, Devon K et al (2022) Quality indicators for the diagnosis and management of primary hyperparathyroidism. JAMA Otolaryngol Head Neck Surg 148:209. 10.1001/jamaoto.2021.3858 34989783 10.1001/jamaoto.2021.3858 PMC8739767

[2] Lappas D, Noussios G, Anagnostis P, Adamidou F, Chatzigeorgiou A, Skandalakis P (2012) Location, number and morphology of parathyroid glands: results from a large anatomical series. Anat Sci Int 87(3):160–164. 10.1016/j.amjsurg.2018.12.074 22689148 10.1007/s12565-012-0142-1

[3] Taterra D, Wong LM, Vikse J, Sanna B, Pękala P, Walocha J et al (2019) The prevalence and anatomy of parathyroid glands: a meta-analysis with implications for parathyroid surgery. Langenbecks Arch Surg 404:63–70. 10.1007/s00423-019-01751-8 30762091 10.1007/s00423-019-01751-8 PMC6394670

[4] Tay D, Das JP, Yeh R (2021) Preoperative localization for primary hyperparathyroidism: a clinical review. Biomedicines 9(4):390. 10.3390/biomedicines9040390 33917470 10.3390/biomedicines9040390 PMC8067482

[5] Hillyar CR, Rizki H, Begum R, Gupta A, Nagabhushan N, Lee PH et al (2022) A retrospective cohort study of the utility of ultrasound, 99mTc-sestamibi scintigraphy, and four-dimensional computed tomography for pre-operative localization of parathyroid disease to facilitate minimally invasive parathyroidectomy. Cureus 14(1):e21177. 10.7759/cureus.21177 35165625 10.7759/cureus.21177 PMC8837380

[6] Fang S, Zhu QL, Liu YM, Zhang ZH, Wang O, Xing XP et al (2024) Localization of ectopic hyperparathyroidism: ultrasound versus 99mTc-sestamibi, 4-dimensional computed tomography, and 11C-choline positron emission tomography/computed tomography. Endocr Pract 30(3):239–245. 10.1016/j.eprac.2023.12.011 38122932 10.1016/j.eprac.2023.12.011

[7] Lee S-W, Shim SR, Jeong SY, Kim S-J (2021) Direct comparison of preoperative imaging modalities for localization of primary hyperparathyroidism: a systematic review and network meta-analysis. JAMA Otolaryngol Head Neck Surg 147:692–706. 10.1001/jamaoto.2021.0915 34081083 10.1001/jamaoto.2021.0915 PMC8176390

[8] Blanco-Saiz I, Goñi-Gironés E, Ribelles-Segura MJ, Salvador-Egea P, Díaz-Tobarra M, Camarero-Salazar A et al (2023) Preoperative parathyroid localization. Relevance of MIBI SPECT-CT in adverse scenarios. Endocrinología, Diabetes y Nutrición 70:35–44. 10.1016/j.endien.2022.11.025 37268356 10.1016/j.endien.2022.11.025

[9] Gowrishankar SV, Bidaye R, Das T, Majcher V, Fish B, Casey R et al (2023) Intrathyroidal parathyroid adenomas: Scoping review on clinical presentation, preoperative localization, and surgical treatment. Head Neck 45:706–20. 10.1002/hed.27287 36563301 10.1002/hed.27287 PMC10108101

[10] Van den Bruel A, Bijnens J, Van Haecke H, Vander Poorten V, Dick C, Vauterin T et al (2023) Preoperative imaging for hyperparathyroidism often takes upper parathyroid adenomas for lower adenomas. Sci Rep 13:7568. 10.1038/s41598-023-32707-0 37160895 10.1038/s41598-023-32707-0 PMC10169799

[11] Adarve Castro A, Domínguez Pinos D, Soria Utrilla V, O’Farrell del Campo JA, Sendra Portero F, Ruiz-Gómez MJ (2024) Update in imaging tests used for the localization of parathyroid pathology. Radiol Engl Ed 66:236–47. 10.1016/j.rxeng.2023.04.006 10.1016/j.rxeng.2023.04.006 38908885

[12] Fong HRC, Zilbermint M (2024) Navigating diagnostic challenges in ectopic parathyroid adenomas: a case report. Cureus 16(9):e68637. 10.7759/cureus.68637 39371798 10.7759/cureus.68637 PMC11452360

[13] Oner M, Hacim NA (2022) Is confirmation of parathyroid tissue by frozen section superior to localization of solitary parathyroid adenoma using intraoperative gamma probe survey? A retrospective cohort study. Acta Endocrinol (Buchar) 18(4):452–457. 10.4183/aeb.2022.452 37152884 10.4183/aeb.2022.452 PMC10162819

[14] Li J, Vasilyeva E, Hiebert J, Britton H, Walker B, Wiseman SM (2019) Limited clinical utility of intraoperative frozen section during parathyroidectomy for treatment of primary hyperparathyroidism. Am J Surg 217(5):893–898. 10.1016/j.amjsurg.2019.01.032 30771863 10.1016/j.amjsurg.2019.01.032

[15] Westra WH, Pritchett DD, Udelsman R (1998) Intraoperative confirmation of parathyroid tissue during parathyroid exploration: a retrospective evaluation of the frozen section. Am J Surg Pathol 22:538. 10.1097/00000478-199805000-00003 9591722 10.1097/00000478-199805000-00003

[16] El-Bolkainy T, Rabie A, Zain M, El-Bolkainy N (2019) El-Bolkainy N. Intraoperative identification of parathyroid tissue: a comparative validity study of frozen section, cytology, and reflected-light diagnostic methods. Egypt J Pathol 39:53. 10.4103/EGJP.EGJP_8_19

[17] Maser C, Kohlbrenner AH, Dirks R (2020) Use of indocyanine green and fluorescence angiography in parathyroid surgery: a feasibility study. Surg Innov 27:587–93. 10.1177/1553350620956437 32892716 10.1177/1553350620956437

[18] Mahmut Z, Zhang C, Ruan F, Shi N, Zhang X, Wang Y, Zheng X, Tang Z, Dong B, Gao D, Sun J (2023) Medical applications and advancement of near infrared photosensitive indocyanine green molecules. Molecules 28(16):6085. 10.3390/molecules28166085 37630337 10.3390/molecules28166085 PMC10459369

[19] Capasso I, Cucinella G, Volcheck G, McGree M, Fought AJ, Chuzhyk O et al (2024) Let go of the myth: safety of indocyanine green for sentinel lymph node mapping in endometrial cancer. Int J Gynecol Cancer 34:80–87. 10.1136/ijgc-2023-004918 38088181 10.1136/ijgc-2023-004918

[20] Rahate NP, Kapse A, Rahate PV, Nimbhorkar SP (2023) The wonder dye: uses and implications of indigocyanine green in various surgeries. Cureus 15(10):e46722. 10.7759/cureus.46722 38021982 10.7759/cureus.46722 PMC10630983

[21] Zaidi N, Bucak E, Okoh A, Yazici P, Yigitbas H, Berber E (2016) The utility of indocyanine green near infrared fluorescent imaging in the identification of parathyroid glands during surgery for primary hyperparathyroidism. J Surg Oncol 113(7):771–774. 10.1002/jso.24240 27039880 10.1002/jso.24240

[22] Faderani R, Yassin AM, Brady C, Caine P, Nikkhah D (2023) Versatility of indocyanine green (ICG) dye in microsurgical flap reconstruction. J Plast Reconstr Aesthet Surg 76:118–20. 10.1016/j.bjps.2022.11.025 36516502 10.1016/j.bjps.2022.11.025

[23] Moreno-Llorente P, Pascua-Solé M, García-Barrasa A, Muñoz-de-Nova JL (2023) Indocyanine green (ICG) angiography-guided thyroidectomy: description of surgical technique. Front Surg 10:1217764. 10.3389/fsurg.2023.1217764 37529659 10.3389/fsurg.2023.1217764 PMC10388241

[24] Urciuoli I, Pernazza G (2024) Indocyanine green–enhanced fluorescence-guided surgery: lymphatic navigation, perfusion evaluation and future perspective s. In: Ceccarelli G, Coratti A (eds) Robotic surgery of colon and rectum. Updates in Surgery, Springer, Cham, pp 189–198. 10.1007/978-3-031-33020-9_24

[25] Khalaf MH, Abdelrahman H, El-Menyar A, Afifi I, Kloub A, Al-Hassani A et al (2024) Utility of indocyanine green fluorescent dye in emergency general surgery: a review ofthe contemporary literature. Front Surg [Internet]. [cited 2024 Dec 2];11. Availablefrom: 10.3389/fsurg.2024.1345831 PMC10899482 38419940

[26] Rossi L, De Palma A, Papini P, Chicas Vasquez M, Cetani F, Ambrosini CE, Materazzi G (2024) Near-infrared autofluorescence pattern in parathyroid gland adenoma. Surg Endosc 38(11):6930–6937. 10.1007/s00464-024-11314-8 39382656 10.1007/s00464-024-11314-8 PMC11525238

[27] Rupp GE, Barba P, Goldhaber NH, Hu J, Bouvet M (2023) Indocyanine green fluorescence guided resection of parathyroid adenoma of the carotid sheath: a case report and review of the literature. Gland Surg. 10.21037/gs-22-589 10.21037/gs-22-589 PMC10186167 37200930

[28] Zhang D, Sun H, Frattini F, Kim HY, Wu CW, Donatini G et al (2022) Use of indocyanine green fluorescence during total thyroidectomy to identify parathyroid glands and prevent hypoparathyroidism. Surg Technol Int 43:77–82. 10.52198/23.sti.43.gs1741 38237113 10.52198/23.STI.43.GS1741

[29] DeLong JC, Ward EP, Lwin TM, Brumund KT, Kelly KJ, Horgan S, Bouvet M (2018) Indocyanine green fluorescence-guided parathyroidectomy for primary hyperparathyroidism. Surgery 163(2):388–392. 10.1016/j.surg.2017.08.018 29129358 10.1016/j.surg.2017.08.018 PMC11060843

[30] Di Meo G, Karampinis I, Gerken A, Lammert A, Pellicani S, Nowak K (2021) Indocyanine green fluorescence angiography can guide intraoperative localization during parathyroid surgery. Scand J Surg 110(1):59–65. 10.1177/1457496919877581 31554490 10.1177/1457496919877581

[31] Rudin AV, McKenzie TJ, Thompson GB, Farley DR, Lyden ML (2019) Evaluation of parathyroid glands with indocyanine green fluorescence angiography after thyroidectomy. World J Surg 43:1538–1543. 10.1007/s00268-019-04909-z 30659346 10.1007/s00268-019-04909-z

[32] Gálvez-Pastor S, Torregrosa NM, Ríos A, Febrero B, González-Costea R, García-López MA et al (2019) Prediction of hypocalcemia after total thyroidectomy using indocyanine green angiography of parathyroid glands: a simple quantitative scoring system. Am J Surg 218:993–999. 10.1016/j.amjsurg.2018.12.074 30665612 10.1016/j.amjsurg.2018.12.074

[33] Akgun E, Berber E (2024) Near-infrared autofluorescence signatures of single- vs multigland disease in primary hyperparathyroidism. JAMA Otolaryngol Head Neck Surg 150:979–85. 10.1001/jamaoto.2024.3095 39325445 10.1001/jamaoto.2024.3095 PMC11428033

[34] Lee SM, Dedhia PH, Shen C, Phay JE (2022) Smaller parathyroids have higher near-infrared autofluorescence intensity in hyperparathyroidism. Surgery 172(4):1114–1118. 10.1016/j.surg.2022.06.027 35981919 10.1016/j.surg.2022.06.027

[35] Wiseman SM, Saleh N, Tootooni A, Eshraghi P, Jama R, Saleh S (2021) Parathyroid identification during thyroid and parathyroid operations: a pilot study evaluating a novel low cost autofluorescence based device. Am J Surg 221(6):1150–1158. 10.1016/j.amjsurg.2021.03.005 33745690 10.1016/j.amjsurg.2021.03.005

[36] Badii B, Staderini F, Foppa C, Tofani L, Skalamera I, Fiorenza G et al (2017) Cost–benefit analysis of the intraoperative parathyroid hormone assay in primary hyperparathyroidism. Head Neck. 10.1002/hed.24567 27557453 10.1002/hed.24567

[37] Morris LF, Zanocco K, Ituarte PHG, Ro K, Duh Q-Y, Sturgeon C et al (2010) The value of intraoperative parathyroid hormone monitoring in localized primary hyperparathyroidism: a cost analysis. Ann Surg Oncol 17:679. 10.1245/s10434-009-0773-1 19885701 10.1245/s10434-009-0773-1 PMC2820694

